# Explainable machine learning to predict root biomass of field crops using UAV multispectral data

**DOI:** 10.3389/fpls.2026.1839186

**Published:** 2026-05-22

**Authors:** Ruiping Shan, Guofang Wang, Shujie Jia, Xinran Liang, FuZhong Li, Wuping Zhang

**Affiliations:** 1 Faculty of Software Technologies, Shanxi Agricultural University, Jinzhong, China; 2 Key Laboratory of Sustainable Dryland Agriculture of Shanxi Province, Taiyuan, China; 3 College of Resources and Environment, Shanxi Agricultural University, Jinzhong, China

**Keywords:** crop phenotyping, machine learning, root biomass, SHAP analysis, UAV multispectral

## Abstract

Understanding below-ground biomass dynamics is essential for improving crop performance in water-limited regions. Yet field-scale root monitoring remains constrained by destructive and labor-intensive sampling. This study presents explainable machine learning models to estimate root biomass of maize, millet, and sorghum using UAV multispectral imagery and key canopy phenotypic traits. Across 405 samples collected during the 2024 growing season, eight algorithms were evaluated, among which Random Forest and XGBoost achieved the highest predictive accuracy (R² = 0.763 for millet, 0.688 for maize, and 0.659 for sorghum). SHAP analysis revealed that leaf area was the dominant predictor across all crops, with 2–3 times greater influence than other traits, while leaf water content and chlorophyll-related parameters exhibited species-specific effects associated with drought adaptation. Under the conditions tested, these results suggest that UAV-based multispectral phenotyping, combined with interpretable machine learning, can enable non-destructive estimation of root biomass at the field scale. Within the limits of this single−site, single−season study, the approach demonstrates potential for large−scale root phenotyping and for supporting crop improvement in semi−arid regions. We quantify a 15−25% reduction in R² relative to above−ground trait prediction, which we term the ‘cost of indirect inference’—highlighting the inherent challenge of estimating below−ground biomass from canopy−level data. These findings offer insights for precision agriculture, subject to broader validation.

## 1 Introduction

Global crop production is increasingly constrained by water scarcity and climatic variability, particularly in dryland agricultural regions (Lynch, 2013). Root systems govern water and nutrient uptake and play a central role in crop adaptation under stress (Fiorani and Schurr, 2013). Understanding root development across species is therefore important for improving drought tolerance and designing efficient irrigation strategies. However, field-scale quantification of root biomass remains challenging. Conventional methods such as soil coring and excavation are destructive, labor-intensive, and unsuitable for repeated measurements over large areas (Pierret et al., 2013), creating a persistent gap between root phenotyping needs and practical monitoring tools.

Advances in remote sensing have notably improved the estimation of above-ground traits, including biomass, canopy structure, chlorophyll content, and water status (Sishodia et al., 2020; Giannico et al., 2024). Yet extending these techniques to below-ground biomass remains poorly explored. Existing studies generally infer root traits indirectly from canopy reflectance or stress responses (Bendig et al., 2015; Yue et al., 2017; Zheng et al., 2019), and most have been limited to single species, controlled environments, or small plots. These efforts often rely on empirical relationships without clarifying which canopy traits drive root variation, limiting biological interpretability. A systematic, cross-crop evaluation of the link between UAV-derived phenotypes and root biomass is still lacking.

Different remote sensing platforms have been explored for root-related applications. Satellite products map root-zone soil moisture at regional scales but lack spatial resolution for field-level use (Zhu et al., 2017). Airborne hyperspectral imaging remains costly for routine monitoring (Zarco-Tejada, 2000; Zarco-Tejada et al., 2012). UAV-based multispectral systems offer a flexible compromise, providing high spatial resolution and rapid deployment at the field scale (Poorter et al., 2012). While UAVs have been widely used to predict above-ground traits, their application to root biomass prediction is still limited, particularly in dryland cereal cropping systems.

The functional equilibrium hypothesis states that plants dynamically adjust biomass allocation between shoots and roots to optimize resource acquisition (Brouwer, 1962). However, its use for operational root monitoring is constrained by the complexity of mechanistic models such as APSIM. Data-driven approaches complement these models by capturing empirical relationships directly from observations, provided they are combined with appropriate interpretability tools.

Machine learning algorithms such as Random Forest and XGBoost have shown good performance in modeling nonlinear spectral–trait relationships (Breiman, 2001; Chen and Guestrin, 2016). Explainable learning tools, including SHAP, provide quantitative insight into feature contributions (Lundberg and Lee, 2017). Nevertheless, applications of explainable ML to below-ground traits remain scarce, and the interactions between canopy phenotypes and root biomass across different cereal species are not yet well characterized.

Millet, sorghum, and maize are major C4 crops widely cultivated in semi-arid regions, each exhibiting distinct patterns of canopy development and root system architecture (Wasson et al., 2012). These contrasting strategies make the three crops suitable for evaluating species-specific shoot–root relationships under UAV-assisted phenotyping.

This study was conducted at a single site during one growing season, with a total of 405 samples (135 per crop). Detailed information on field conditions, soil properties, and climate is provided in the Materials and Methods section.

Given the single-site, single-season design of this experiment, our results should be interpreted as preliminary and specific to the conditions studied. In this context, the objectives of this study were: (1) to develop machine-learning models for predicting root biomass of maize, millet, and sorghum using UAV-derived multispectral phenotypic parameters; and (2) to identify the key above-ground features driving root variation through SHAP and partial dependence analyses, thereby improving the interpretability and physiological relevance of UAV-based root biomass estimation.

## 2 Materials and methods

### 2.1 Study site description

**Figure 1:**
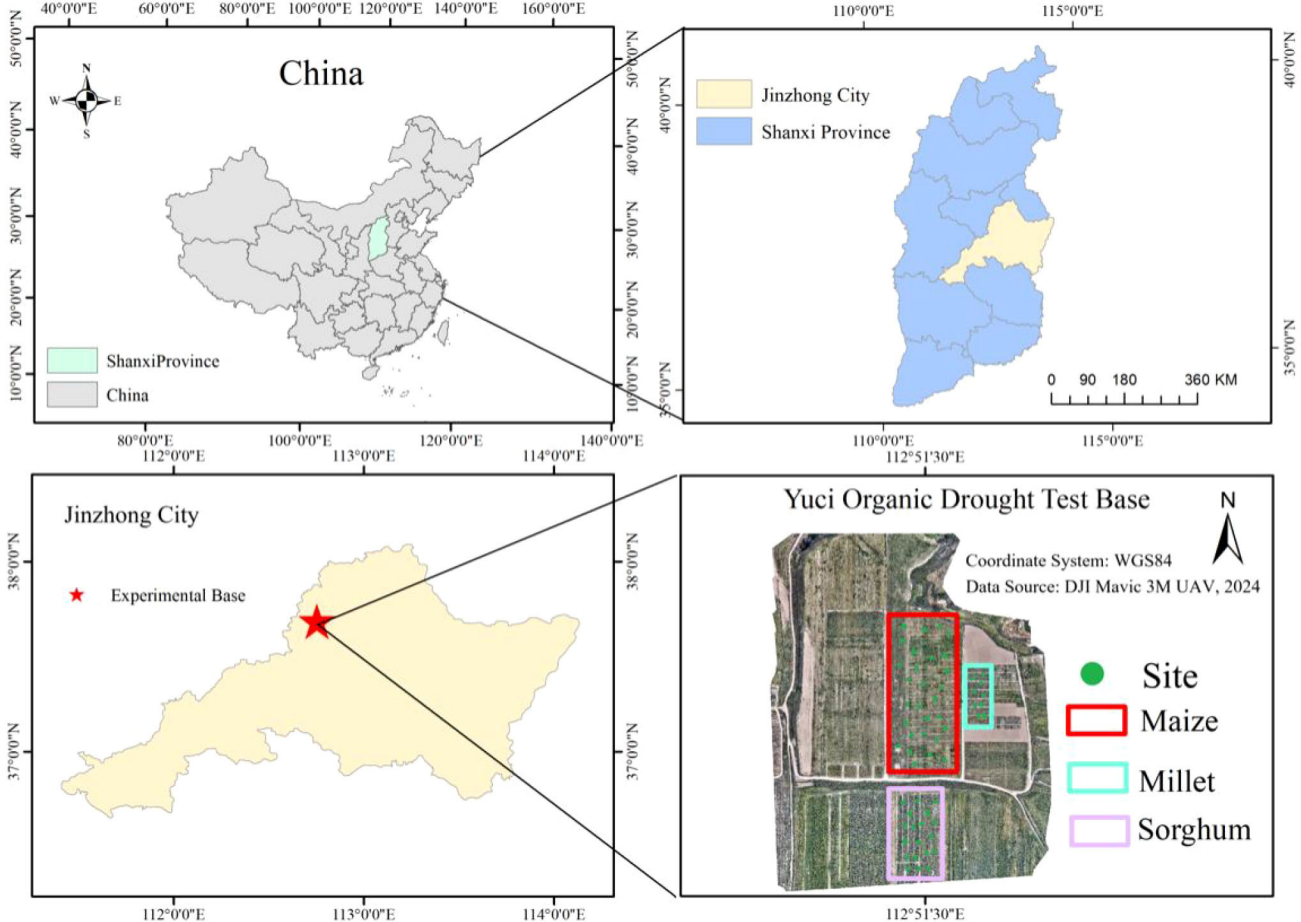
Multi-scale map showing the location of the experimental site. The study was conducted in Yuci District, Jinzhong City, Shanxi Province, China (N37°51’, E112°45’).

The experimental site was located in the organic dry farming research area of Yuci District, Jinzhong City, Shanxi Province (N37°51’, E112°45’) (**Figure 1**). The region has a temperate continental semi-arid climate. Annual sunshine hours range from 2000 to 3000 hours, and annual evaporation ranges from 1500 to 2300 mm. Elevation varies between 767 and 1777 m above sea level. The frost-free period lasts 120 to 220 days. The mean annual temperature is approximately 9.8 °C. Annual precipitation mainly occurs from July to September, totaling 418 to 483 mm (China Meteorological Data Service Center, 2024; Deng et al., 2025). All climatic data used in this study were obtained from an *in-situ* micro-meteorological station deployed within the experimental site, which continuously recorded temperature, precipitation, evaporation, and solar radiation throughout the growing season. The soil type is a mixture of yellow cinnamon soil and loamy soil, with an organic matter content of 17.6 g kg^-^¹, total nitrogen content of 0.98 g kg^-^¹, and a field water-holding capacity of 21% (v/v) at the 0–30 cm depth (Li et al., 2025). From May to October 2024, the experiment was conducted under a strict rain-fed regime with no irrigation, relying entirely on natural precipitation. This provided an ideal setting for studying the root development characteristics and the synergistic relationship between shoot-root parts of maize, millet, and sorghum in a semi-arid environment.

### 2.2 Data collection and processing

#### 2.2.1 Collection and processing of multispectral data

In this study, multispectral data were collected using a DJI Mavic 3M UAV during critical growth stages of maize, millet, and sorghum. The flight altitude was set to 65 m, and data were captured between 10:00 and 12:00 local time under clear weather conditions (**Table 1**). The forward and overlap rates were 70% and 80%, respectively. The UAV-integrated spectral system acquired reflectance data in four bands: red (650 ± 16 nm), green (560 ± 16 nm), red-edge (730 ± 16 nm), and near-infrared (860 ± 26 nm).UAV flights were conducted at critical growth stages (**Table 2**), which are defined as key developmental periods when significant changes in above-ground phenology and root development occur. For maize, these stages included seedling (V3-V5), jointing (V6-V8), tasseling (VT), silking (R1), grain-filling (R3-R4), and maturity (R6). For millet and sorghum, corresponding stages were identified based on field observations of canopy development, including tillering, booting, heading, flowering, grain-filling, and maturity stages. Flight timing was determined by monitoring crop phenology in the field, ensuring that each flight captured distinct physiological phases characterized by different leaf area, biomass accumulation, and water status patterns.

**Table 1:** UAV flight dates and corresponding growth stages of maize, millet, and sorghum during the 2024 growing season.

Flight date	Maize growth stage	Millet growth stage	Sorghum growth stage
May 26, 2024	Seedling	Seedling	Seedling
June 4, 2024	Seedling	Seedling	Seedling
June 17, 2024	Jointing	Tillering	Jointing
July 2, 2024	Jointing	Tillering	Jointing
July 28, 2024	Tasseling	Booting	Booting
July 30, 2024	Tasseling	Booting	Booting
August 14, 2024	Silking	Heading	Heading
August 15, 2024	Silking	Heading	Heading
August 17, 2024	Silking	Flowering	Flowering
August 24, 2024	Grain-filling	Grain-filling	Grain-filling
September 3, 2024	Grain-filling	Grain-filling	Grain-filling
September 10, 2024	Grain-filling	Grain-filling	Grain-filling
September 19, 2024	Maturity	Maturity	Maturity
September 28, 2024	Maturity	Maturity	Maturity
October 15, 2024	Maturity	Maturity	Maturity

**Table 2:** Technical specifications of the DJI Mavic 3M UAV platform and onboard sensor systems used in this study.

Category	Parameter	Specification
Aircraft Platform	Model	DJI Mavic 3M Multispectral
	Takeoff Weight	1050 g
	Max Flight Time	43 min
	Max Wind Resistance	12 m/s
	Wheelbase	380.1 mm
Multispectral Camera	Sensor Type	1/2.8-inch CMOS
	Effective Pixels	5 Megapixels (per band)
	Spectral Bands	Green (G): 560 ± 8 nm
		Red (R): 650 ± 8 nm
		Red Edge (RE): 730 ± 8 nm
		Near-Infrared (NIR): 860 ± 13 nm

Radiometric calibration was performed using a 0.5 × 1 m standard gray panel (50% reflectance) photographed immediately before and after each flight under the same lighting conditions as image acquisition. The panel was placed horizontally on the ground within the flight area, and images were captured from the same altitude (65 m) as the crop imagery. Pre-flight and post-flight panel images were used to calculate calibration coefficients accounting for changes in solar irradiance during the flight period. The radiometric calibration procedure followed these steps: (1) Raw digital numbers (DN) from panel images were extracted for each spectral band; (2) Calibration coefficients were calculated using the known panel reflectance and measured DN values; (3) Crop imagery was converted from DN to reflectance using the calibration coefficients; (4) Linear interpolation was applied to correct for temporal changes in solar irradiance between pre-flight and post-flight calibrations. This procedure ensured that all spectral data were normalized to consistent reflectance units, enabling comparison across different flight dates and environmental conditions. Calibration uncertainty was not formally quantified in this study. However, based on the consistency of panel reflectance measurements across multiple flights under varying illumination conditions, the uncertainty is expected to be within an acceptable range (approximately 5–10%) for agricultural remote sensing applications. No onboard sunlight sensor was used; illumination changes were corrected using pre−flight and post−flight panel measurements. Vignette correction was not applied, as the central region of interest (plot area) occupies only a small portion of the image where vignetting effects are negligible. Reflectance calibration was performed separately for each of the four multispectral bands (G, R, RE, NIR) using the same standard reference panel. No independent atmospheric correction was performed. Given the low flight altitude (65 m) and clear weather conditions during all flights, atmospheric effects on the multispectral signal are considered negligible. Moreover, the panel−based radiometric calibration procedure described above inherently accounts for any residual atmospheric influences because the reference panel and crop images were acquired under identical atmospheric conditions and at the same distance from the sensor (Aasen et al., 2018).

Image stitching and orthorectification were conducted using DJI Terra, and spectral reflectance was extracted using ArcMap. Multiple vegetation indices were subsequently calculated, and quantitative models were developed to characterize the relationships between above-ground phenotypic parameters and crop root development. Reflectance values corresponding to each ground-measured sampling point were extracted through a standardized spatial matching procedure. After radiometric calibration and orthorectification, the multispectral images were converted into georeferenced reflectance mosaics. Each field sampling location was recorded using a handheld GPS (± 0.5 m accuracy). To account for potential geolocation offsets and pixel-level heterogeneity, a circular buffer with a radius of 0.5 m was generated around each sampling point. The mean reflectance of all pixels within this buffer was then extracted for the red, green, red-edge, and NIR bands and used as the UAV-derived reflectance associated with the corresponding field measurement. Climatic data for the 2024 growing season were obtained from an *in-situ* micro-meteorological station deployed within the experimental site, which continuously recorded temperature, precipitation, evaporation, and solar radiation throughout the study period.

#### 2.2.2 Leaf water content

In this study, Leaf water content (LWC) represents the proportion of water in fresh leaf tissue, which is an important indicator of plant water status and physiological activity. For each crop, three replicate 50 cm × 50 cm sample plots were established, with positions accurately marked using GPS to ensure consistency with the locations of multispectral image data collection. A total of 15 sampling events were conducted throughout the growing season, corresponding to each UAV flight date. At each sampling event, three representative plants were randomly selected from each plot, resulting in 135 samples per crop and 405 samples in total across maize, millet, and sorghum. Sampling points were selected to cover different zones and conditions throughout the crop growth cycle, enhancing the generalizability of the data. Within the sample plots, the water content and coverage of the canopy leaves of the three crops were measured manually. The LWC was determined using the weighing method, where the fresh weight and dry weight of the leaves were recorded to calculate the water content (Equation 1) (Ramadhan et al., 2021).

(1) $$\text{LWC}=\frac{\text{FW}-\text{DW}}{\text{FW}}\times 100\%$$

In this study, in Equation 1, LWC represents the canopy leaf water content, FW denotes the fresh weight of the leaves, and DW stands for the dry weight of the leaves.

#### 2.2.3 Leaf chlorophyll content

The SPAD value is a relative measure of leaf chlorophyll content, which reflects the photosynthetic capacity and nitrogen status of crops. The chlorophyll content of the crop canopy leaves was measured using a portable CM-1000 Chlorophyll Meter. At each sampling point, a representative section of the crop canopy was selected. Measurements were taken on three distinct parts—the leaf tip, middle section, and leaf base—of both the topmost fully expanded leaf (penultimate leaf) and the leaf immediately below it (antepenultimate leaf). Five replicate readings were recorded for each specific leaf part, and the average value was calculated to represent the chlorophyll index (SPAD value) for that particular crop canopy.

#### 2.2.4 Leaf area

**Figure 2:**
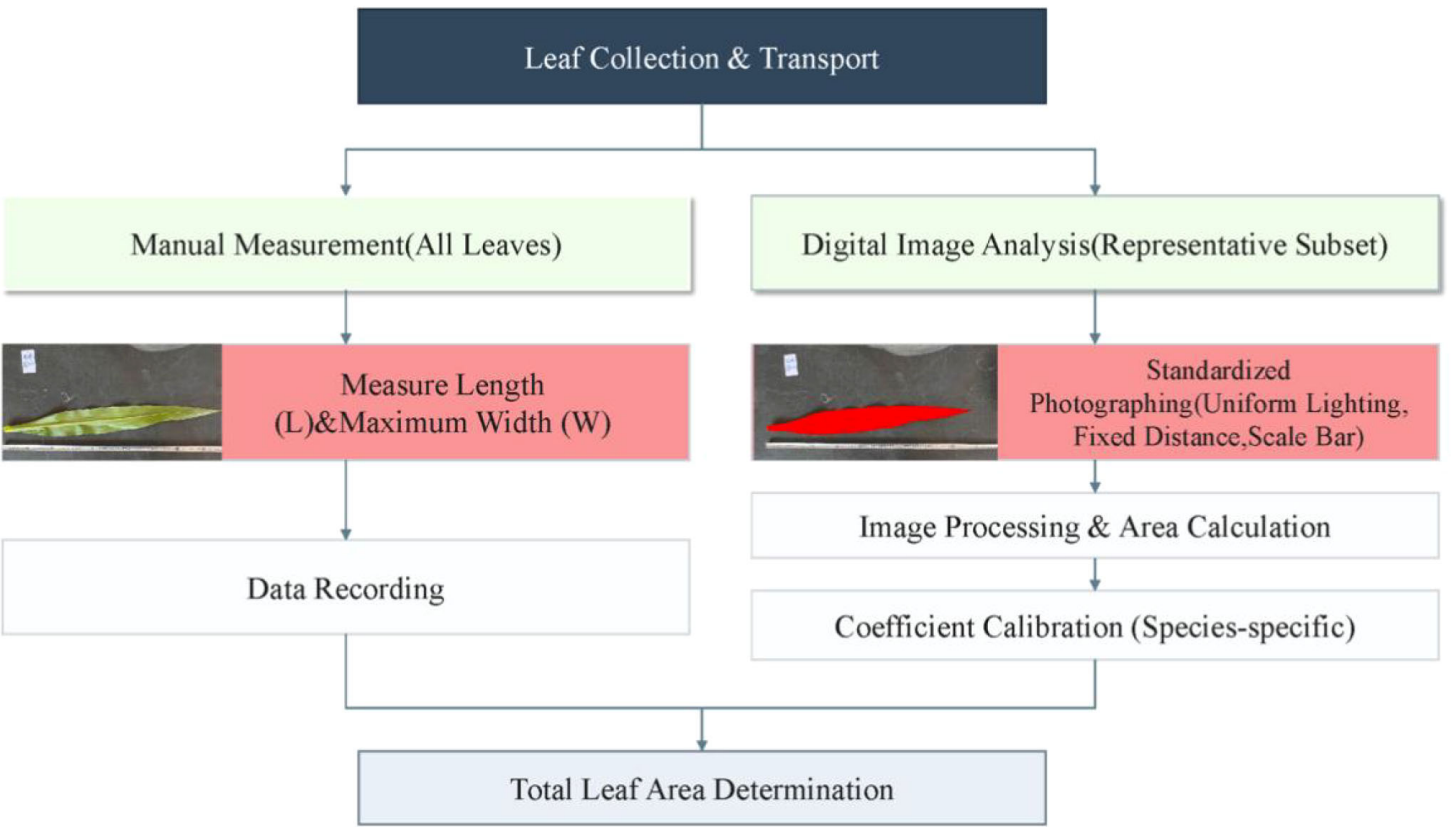
Workflow for leaf area measurement and species-specific coefficient calibration. The process combines manual measurements of leaf length (L) and maximum width (W) with digital image analysis. Representative leaves were photographed with a calibrated scale bar (as shown in the embedded images) to extract the actual leaf area, which was then used to determine the calibration coefficient (k) for maize, millet, and sorghum through linear regression.

Leaf area was determined using a combination of manual measurement and digital image analysis. All leaves from the sampled plants were collected and transported to the laboratory. For each leaf, length (L) and maximum width (W) were measured manually using a ruler. A subset of representative leaves was selected from each sampling event for digital analysis to calibrate species-specific leaf area coefficients. These leaves were photographed under standardized indoor conditions with uniform artificial lighting using a Nikon D3300 DSLR camera (24.2 megapixels, DX-format CMOS sensor, Nikon Corporation, Tokyo, Japan). The camera was positioned perpendicular to the leaf surface at a fixed distance, and a calibrated scale bar was included in each image to ensure accurate measurements. Leaf area was extracted from the photographs using ArcGIS software by manually digitizing leaf boundaries. The digitally measured leaf areas were used to establish the relationship between manually measured dimensions (L × W) and actual leaf area through linear regression (**Figure 2**), yielding species-specific coefficients of k = 0.75 for maize, k = 0.60 for millet, and k = 0.68 for sorghum. This species−specific calibration represents a methodological strength of our study, as it avoids the use of generic shape coefficients from the literature that may not accurately reflect local growing conditions or leaf morphology. The final leaf area for all leaves was calculated using the formula: Leaf Area = L × W × k.

#### 2.2.5 Leaf area index

Leaf area index (LAI) is defined as the total one-sided area of leaf tissue per unit ground surface area, serving as a key indicator of canopy structure and light interception capacity. Following the completion of leaf area measurement, the calibrated leaf area data were combined with the measured length and width data, resulting in a leaf area coefficient of 0.6 for millet. Furthermore, the crop planting density within a 0.25 m² area surrounding each sampling point was recorded in the field. The LAI was then calculated using (Equation 2) (Daughtry et al., 1983).

(2) $$\text{LAI}=\text{k}*\rho *\sum_{\text{i}=1}^{\text{n}}({\text{L}}_{\text{i}}{*\text{W}}_{\text{i}})*10^{-4}$$

In Equation 2, k is the leaf area coefficient (dimensionless; 0.6 for millet), ρ is the planting density (plants m^-^²), n is the number of leaves per plant, L_i_ ​and W_i_​ are the length (cm) and width (cm) of the i-th leaf, and the factor 10^−4^ converts cm² to m². The calculation steps are as follows: (1) measure length and width of each leaf on a sampled plant; (2) compute single-leaf area as L_i_ × W_i_ × k; (3) sum over all leaves to obtain leaf area per plant; (4) multiply by planting density to obtain LAI. This method ensures that LAI is directly derived from leaf dimensions, planting density, and the species-specific shape coefficient.

#### 2.2.6 Above-ground biomass

Above-ground biomass (AGB) refers to the total dry weight of all plant organs above the soil surface, representing the cumulative result of photosynthetic carbon assimilation. During each sampling event, three uniformly growing plants were randomly selected from the sampling area within each experimental plot. The plants were carefully excavated with their entire root systems, after which the roots were excised. The above-ground sections were thoroughly rinsed with clean water, and the non-panicle parts and panicles (when present after the heading stage) were separated and placed into individual sample bags. The sample bags were then placed in an oven and exposed to 105 °C for 30 minutes to deactivate enzymes. Subsequently, the temperature was reduced to 80 °C for continued drying until a constant weight was achieved. After the drying process was complete, the dry biomass of each sample was weighed and recorded separately. The above-ground dry biomass per unit area (g/m²) was then calculated based on the crop planting density.

#### 2.2.7 Root biomass

This study employed the excavation and root washing method to determine the root biomass of maize, millet, and sorghum. During key growth stages, synchronized with the collection of above-ground phenotypic parameters, three representative plants were randomly selected for root sampling from each experimental plot. A 40 cm × 40 cm × 40 cm soil monolith, centered on the plant, was excavated. The plant was carefully separated from the soil to preserve the integrity of the entire root system. The collected root samples were transported to the laboratory, where they were initially rinsed with tap water, followed by meticulous washing over a 0.5 mm mesh sieve to remove all adhering soil particles while ensuring root integrity. The cleaned root samples were placed in pre-labeled paper bags and dried in a constant-temperature oven at 75 °C until a constant weight was achieved (approximately 48 hours). The dry weight was then measured using a precision balance (accuracy: 0.001g) and recorded as the root biomass. Each sample was measured three times, and the average value was taken as the final data (Boehm, 1979). It should be noted that for crops with rooting depth potentially exceeding 40 cm (e.g., maize and sorghum), the excavated soil monolith captures only a portion of the total root system; therefore, the measured root biomass represents partial rather than total root biomass.

#### 2.2.8 Development phases and root growth dynamics

This study For the three C4 cereals examined in this study—maize, millet, and sorghum—the developmental cycle can be broadly divided into seedling, vegetative, reproductive, and grain-filling phases. Shoot growth during the vegetative stage is characterized by rapid leaf expansion, increasing LAI, and steady accumulation of AGB. Root development follows a parallel but distinct trajectory. In the early seedling stage, crops establish a primary root axis and several seminal roots. During the vegetative phase, root elongation and lateral branching accelerate, with most root biomass accumulating from the jointing to booting stages. Root proliferation typically slows after flowering as carbon allocation shifts toward reproductive structures.

For maize and sorghum, root growth peaks between V6–V12 and continues at a reduced rate until shortly after anthesis, whereas millet shows a more synchronous pattern between tillering and panicle initiation. These developmental features are essential for interpreting the phenotypic traits used in our modeling framework. UAV flights were conducted during periods when both canopy traits and root biomass exhibited strong growth dynamics, ensuring that vegetation indices captured meaningful physiological differences among plants. Similarly, root sampling was conducted during this period of active root development to ensure that the measured root biomass represented the cumulative growth achieved up to that specific phenological stage.

### 2.3 Vegetation indices

Vegetation indices (VIs), serving as key spectral parameters that reflect the physio-ecological characteristics of crops, can effectively characterize the status of above-ground growth. They provide a key bridge for establishing quantitative relationships between above-ground phenotypes and below-ground root development. Given that different VIs exhibit varying sensitivities to specific crop parameters, this study calculated thirteen typical VIs—NDVI, RDVI, NLI, GNDVI, RVI, SAVI, NDGI, DVI, OSAVI, GI, MSR, GRVI, and CLgreen—based on the band data collected by the UAV multispectral sensor (Jordan, 1969; Rouse et al., 1974; Huete, 1988; Goel and Qin, 1994; Elvidge and Chen, 1995; Roujean and Breon, 1995; Gitelson et al., 1996, Gitelson et al., 2005; Rondeaux et al., 1996; Zarco-Tejada, 2000; Haboudane, 2004; Motohka et al., 2010). This comprehensive set of indices was used to characterize the canopy attributes of maize, millet, and sorghum, aiming to identify the optimal spectral indicators for delineating the relationship between shoot-root development. The specific calculation formulas are listed in **Table 3**.

**Table 3:** Vegetation indices calculated from UAV multispectral reflectance data, including their formulas and corresponding scientific references.

Vegetation indices	Formula
Normalized Difference Vegetation Index(NDVI)	NDVI=(NIR−RED)/(NIR+RED)
Renormalized Difference Vegetation Index(RDVI)	RDVI=(NIR−RED/sqrt(NIR+RED)
Normalized Lenticel Index(NLI)	$\text{NLI}=(NIR^{2}-\text{RED})/(NIR^{2}+\text{GREEN})$
Green Normalized Difference Vegetation Index(GNDVI)	GNDVI=(NIR−GREEN)/(NIR+GREEN)
Ratio Vegetation Index(RVI)	RVI=NIR/RED
Soil Adjusted Vegetation Index(SAVI)	SAVI=1.5(NIR−RED)/(NIR+RED+0.5)
Normalized Difference Greenness Index(NDGI)	NDGI=(GREEN−RED)/(GREEN+RED)
Difference Vegetation Index(DVI)	DVI=NIR−Red
Optimized Soil Adjusted Vegetation Index(OSAVI)	OSAVI=1.16(NIR−RED/(NIR+RED+0.16))
Greenness Index(GI)	GI=GREEN/RED
Modified Simple Ratio(MSR)	MSR=(NIR/RED−1)/((NIR/RED+1))×0.5
Green Red Vegetation Index(GRVI)	GRVI=(GREEN−Red)/(GREEN+Red)
Chlorophyll green Index(CLgreen)	CLgreen=(NIR/GREEN)−1

These indices were used as input features for machine-learning models to characterize above-ground phenotypic traits related to crop root development.

### 2.4 Model construction and evaluation

This study employed eight machine-learning algorithms—Linear Regression, LASSO Regression, Ridge Regression, Elastic Net, Random Forest (RF), Gradient Boosting Machine (GBM), Extreme Gradient Boosting (XGBoost), and Support Vector Regression (SVR)—to develop predictive models (Hoerl and Kennard, 1970; Cortes and Vapnik, 1995; Tibshirani, 1996; Breiman, 2001; Friedman, 2001; Zou and Hastie, 2005; James et al., 2013; Chen and Guestrin, 2016). The models were trained using a combination of UAV-derived spectral features and field-measured above-ground traits (e.g., AGB, SPAD, LAI, leaf area, and LWC). The entire dataset was randomly partitioned into five folds for cross−validation. In each fold, 80% of the data (four folds) were used for training and the remaining 20% (one fold) for validation, resulting in a 5−fold cross−validation scheme. The reported R², RMSE, and MAE values in **Table 4** are the averages across the five validation folds; no independent test set was used. No grouping by plot or date was applied during fold creation. We acknowledge that this random fold creation without grouping by plot or date may lead to optimistic performance estimates due to potential information leakage (i.e., samples from the same plot across different growth stages could appear in both training and validation folds). Therefore, the reported performance metrics should be interpreted with caution. For a dataset of this size (405 samples), cross-validation provides more stable and reliable performance estimates than a single train-test split. All algorithms were trained using fixed hyperparameters (**Table 5**); no hyperparameter tuning was performed. Model performance was evaluated using the coefficient of determination (R²) (Equation 3), root mean square error (RMSE)(Equation 4), and mean absolute error (MAE)(Equation 5), where higher R² and lower RMSE/MAE indicate superior predictive ability.

**Table 4:** Performance metrics (R², R²_Std, RMSE, MAE) of the best−performing algorithm for each crop and parameter.

Crop	Parameter	Best algorithm	R²	R²_Std	RMSE	MAE
Maize	AGB	RF	0.723	0.080	143.277	106.798
	SPAD	RF	0.944	0.014	0.409	0.280
	LAI	RF	0.943	0.027	648.975	453.906
	Leaf Area	SVM	0.813	0.062	24.461	14.847
	LWC	RF	0.525	0.205	0.045	0.030
	Root Biomass	RF	0.688	0.205	14.903	8.740
Millet	AGB	RF	0.655	0.087	11.914	9.297
	SPAD	Elastic Net	0.726	0.082	0.727	0.540
	LAI	RF	0.630	0.111	332.625	242.280
	Leaf Area	RF	0.767	0.123	25.842	15.593
	LWC	RF	0.527	0.281	0.050	0.034
	Root Biomass	XGBoost	0.763	0.14	1.944	0.901
Sorghum	AGB	RF	0.468	0.191	112.802	79.666
	SPAD	SVM	0.701	0.164	0.758	0.615
	LAI	Ridge	0.561	0.162	1073.656	880.300
	Leaf Area	SVM	0.710	0.173	31.656	21.335
	LWC	RF	0.670	0.14	0.029	0.021
	Root Biomass	RF	0.659	0.591	17.395	10.789

**Table 5:** Hyperparameter ranges tested for machine learning algorithms.

Algorithm	Parameter	Tested range
RF	n_estimators	50, 100, 200, 300
	max_depth	5, 10, 15, 20, None
	min_samples_split	2, 5, 10
XGBoost	n_estimators	50, 100, 200
	max_depth	3, 5, 7, 10
	learning_rate	0.01, 0.05, 0.1, 0.2
GBM	n_estimators	50, 100, 200
	max_depth	3, 5, 7
	learning_rate	0.01, 0.05, 0.1
SVR	kernel	linear, rbf, poly
	C	0.1, 1, 10, 100
	epsilon	0.01, 0.1, 0.2
LASSO	alpha	0.001, 0.01, 0.1, 1
Ridge	alpha	0.001, 0.01, 0.1, 1, 10
Elastic Net	alpha	0.001, 0.01, 0.1, 1
	l1_ratio	0.2, 0.5, 0.8

Linear Regression models linear associations between predictors and responses. LASSO, Ridge, and Elastic Net introduce L1, L2, or mixed regularization to prevent overfitting and improve generalization. RF constructs an ensemble of decision trees via bootstrap sampling, averaging predictions to capture nonlinear relationships. GBM builds sequential trees to iteratively reduce residuals through gradient-based optimization. XGBoost enhances traditional boosting by incorporating shrinkage, regularization, and column sampling, offering strong performance and computational efficiency. SVR maps inputs into a high-dimensional feature space and fits an ϵ-insensitive regression tube, enabling effective modeling of nonlinear predictor–response patterns.

(3) $$R^{2}=\frac{\sum_{\text{i}=1}^{\text{n}}({\text{x}}_{\text{i}}-\bar{\text{x}})({\text{y}}_{\text{i}}-\bar{\text{y}})}{\sqrt{\sum_{\text{i}=1}^{\text{n}}{\left({\text{Y}}_{\text{i}}-\bar{\text{Y}}\right)}^{2}}\sqrt{\sum_{\text{i}=1}^{\text{n}}{\left({\text{X}}_{\text{i}}-\bar{\text{X}}\right)}^{2}}}$$

(4) $$\text{RMSE}=\sqrt{\frac{1}{\text{n}}\sum_{\text{i}=1}^{\text{n}}{\left({\text{y}}_{\text{i}}-{\hat{\text{y}}}_{\text{i}}\right)}^{2}}$$

(5) $$\text{MAE}=\frac{1}{\text{n}}\sum_{\text{i}=1}^{\text{n}}|{\text{y}}_{\text{i}}-{\hat{\text{y}}}_{\text{i}}|$$

Hyperparameter ranges were selected based on commonly used values in agricultural remote sensing studies and the characteristics of the dataset. For Random Forest, n_estimators ranged from 50 to 300 to ensure sufficient trees for stable predictions while avoiding excessive computational cost. The max_depth range (5, 10, 15, 20, None) was tested to balance model complexity and overfitting risk, with “None” allowing trees to expand until all leaves are pure. The min_samples_split values (2, 5, 10) control the minimum number of samples required to split an internal node, preventing overfitting in smaller datasets. For XGBoost and GBM, n_estimators were limited to 50–200 to prevent overfitting in boosting algorithms. The max_depth ranges (3–10 for XGBoost, 3–7 for GBM) were kept relatively shallow, as boosting methods are more prone to overfitting with deep trees. Learning rates (0.01-0.2) were tested to find the optimal balance between training speed and model accuracy, with lower rates requiring more estimators but potentially achieving better generalization. For SVR, three kernel functions (linear, rbf, poly) were tested to accommodate different data structures. The C parameter (0.1-100) controls the trade-off between maximizing the margin and minimizing training error, while epsilon (0.01-0.2) defines the width of the epsilon-insensitive zone. For regularization-based methods (LASSO, Ridge, Elastic Net), alpha values ranged from 0.001 to 10, spanning from weak to strong regularization. The l1_ratio in Elastic Net (0.2, 0.5, 0.8) determines the balance between L1 and L2 penalties. Vegetation indices calculated from the same spectral bands often exhibit correlations. To address potential multicollinearity issues, this study employed both regularization-based methods (LASSO, Ridge, Elastic Net) and tree-based ensemble algorithms (RF, XGBoost, GBM). Regularization methods automatically reduce the influence of redundant features through penalty terms, while tree-based methods are inherently robust to correlated predictors due to their recursive partitioning structure and random feature sampling strategies. **Table 5** lists the hyperparameter ranges that were initially considered. However, no systematic hyperparameter tuning (e.g., grid search or random search with cross−validation) was performed in this study. Instead, we selected fixed parameter values for each algorithm based on commonly used settings in agricultural remote sensing literature and preliminary trials. Specifically: for Random Forest – n_estimators=200, max_depth=10, min_samples_split=2; for XGBoost – n_estimators=100, max_depth=5, learning_rate=0.1; for GBM – n_estimators=100, max_depth=5, learning_rate=0.05; for SVR – kernel=‘rbf’, C = 10, epsilon=0.1; for LASSO, Ridge, and Elastic Net – alpha=0.1, and for Elastic Net additionally l1_ratio=0.5. These fixed configurations were applied to all models to ensure a fair comparison under identical conditions, focusing on the relative performance of different algorithm families rather than optimizing each algorithm to its individual peak. For XGBoost and GBM, only the most frequently tuned hyperparameters (n_estimators, max_depth, learning_rate) are listed in **Table 5**. Other parameters (e.g., subsample, colsample_bytree, reg_alpha, reg_lambda for XGBoost) were kept at their library default values (subsample=1.0, colsample_bytree=1.0, reg_alpha=0, reg_lambda=1; for GBM, subsample=1.0, etc.) because no exhaustive hyperparameter search was performed.

Two types of models were developed in this study. First, vegetation indices (VIs) were used as input features to estimate above−ground phenotypic traits (AGB, SPAD, LAI, leaf area, LWC) – referred to as the trait inversion models. Second, the five above−ground traits (either manually measured or estimated from VIs) were used as input features to predict root biomass – referred to as the root biomass prediction models. The SHAP analysis reported in the Results section was performed on the root biomass prediction models using the five traits as predictors. No direct combination of VIs and traits was used as input for root biomass prediction.

### 2.5 Models’ explainability

SHAP is an interpretable machine learning method grounded in the concept of Shapley values from cooperative game theory (Lundberg and Lee, 2017). It provides a unified measure of feature importance by calculating the marginal contribution of each feature to the model prediction across all possible feature combinations. To gain an in-depth understanding of the contribution and influence mechanisms of above-ground phenotypic features in predicting root development, this study employed two interpretable machine learning methods: SHAP value analysis and Partial Dependence Plot (PDP) analysis. SHAP analysis, grounded in the concept of Shapley values from cooperative game theory, precisely quantifies the magnitude and direction of each feature’s contribution to the model predictions. It not only identifies key variables but also reveals complex interaction effects between them. Specifically, we calculated SHAP values using both the TreeExplainer (for tree-based models) and the KernelExplainer (for other model types), with the results visualized through SHAP summary plots and dependence plots to illustrate the influence patterns of individual features.

Partial Dependence Analysis, on the other hand, evaluates the marginal relationship between a single feature and the predicted outcome by controlling for the effects of all other variables (Greenwell, 2017). The PDP curves illustrate how the predicted root biomass changes with a specific feature while other features are held constant, facilitating the identification of non-linear relationships and potential threshold effects.

The combined application of these two analytical approaches enabled both a quantitative assessment of the relative importance of the five key phenotypic indicators—AGB, SPAD value, LAI, leaf area, and LWC—in predicting root biomass, and an exploration of the patterns through which these above−ground features are associated with root development. This provides empirical support for understanding the shoot−root relationships in crops, although the observed associations do not imply direct causal mechanisms.

SHAP and PDP analyses were performed on the best-performing root biomass prediction models for each crop, using the five above-ground phenotypic parameters (leaf area, LWC, AGB, LAI, and SPAD) as input features. The analyses were conducted separately for each crop using their optimal models: XGBoost for millet and Random Forest for maize and sorghum.

### 2.6 Computational environment

All analyses were conducted in Python (version 3.10). Data preprocessing was performed using NumPy and Pandas, and machine learning modeling was implemented with Scikit-learn. Gradient boosting models were trained using XGBoost and LightGBM. Visualization was carried out using Matplotlib and Seaborn, while model explainability was evaluated through SHAP and PDPbox.

## 3 Results

### 3.1 Model performance overview

Model performance for predicting above-ground phenotypic parameters and root biomass varied by crop and parameter (**Table 4**). Random Forest emerged as the best-performing algorithm in 12 out of 18 cases, demonstrating its robustness across different prediction tasks. Support Vector Machine performed best for 3 cases, while XGBoost, Elastic Net, and Ridge each achieved optimal performance in one case.

Model performance was evaluated using 5−fold cross−validation. RMSE and MAE values differ substantially across parameters due to inherent differences in measurement units and value ranges (e.g., AGB in kg/ha vs. LWC as a proportion). Therefore, R² was used as the primary metric for cross−parameter comparisons, as it is scale−independent. Formal statistical tests for comparing algorithms were not conducted. However, the ranking of algorithms was consistent across cross−validation folds, suggesting stable performance differences. The standard deviations of R² (R²_Std) across folds are reported in **Table 4**, ranging from 0.014 to 0.591. These values indicate that prediction stability varies by crop and parameter, with generally higher stability for well−predicted traits (e.g., SPAD in maize, R²_Std = 0.014) and lower stability for traits with lower accuracy (e.g., sorghum root biomass, R²_Std = 0.591). Fold−wise confidence intervals are not reported for brevity but are available upon request.

### 3.2 Above-ground trait inversion models

**Figure 3:**
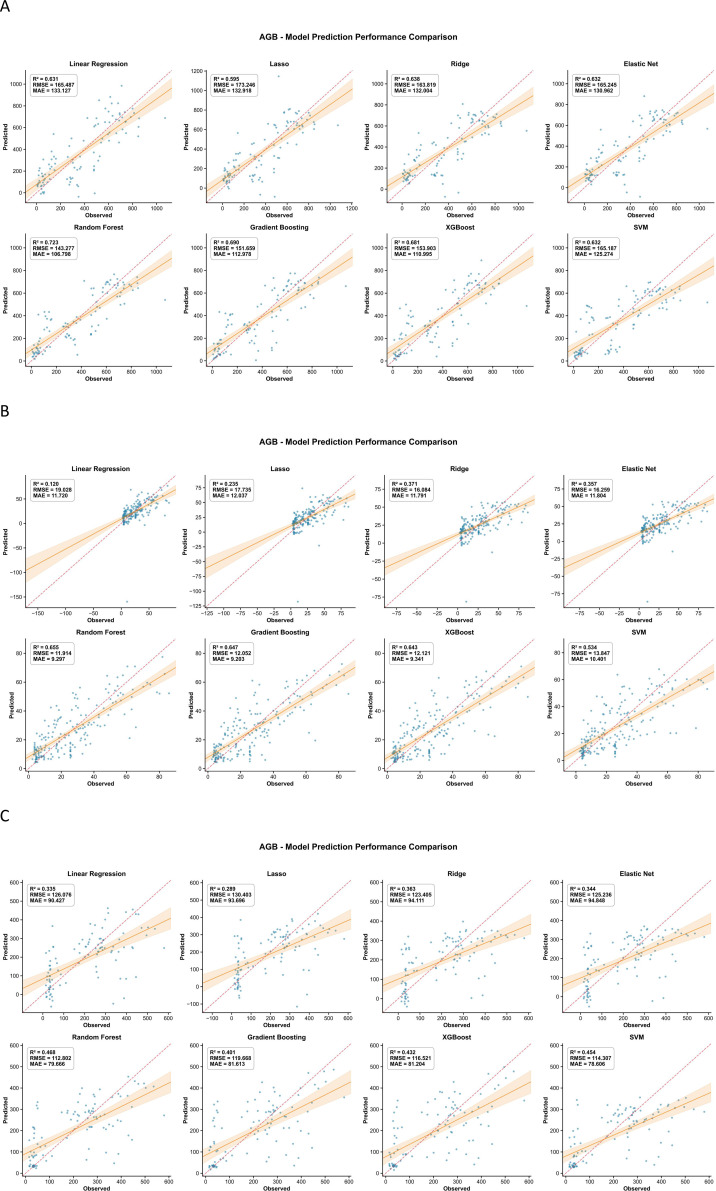
Comparison of predicted versus measured AGB from the inversion models for the three crops. **(A)** Represents maize, **(B)** represents millet, and **(C)** represents sorghum.

**Figure 4:**
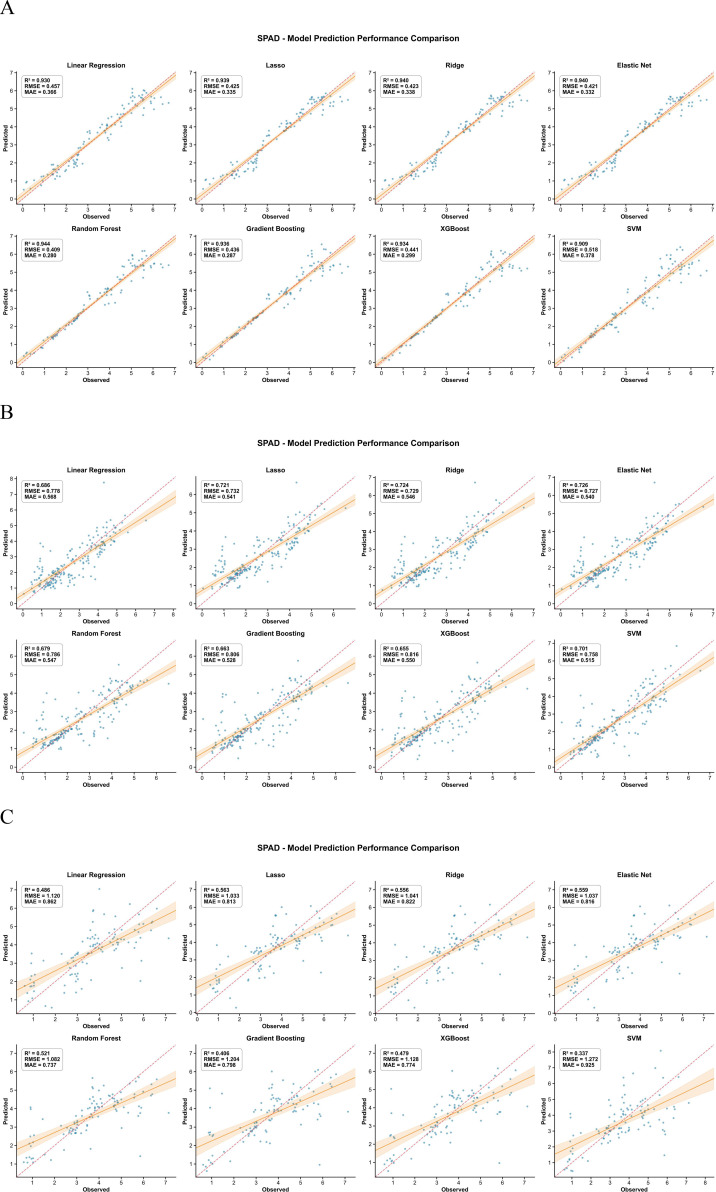
Comparison of predicted versus measured SPAD values from the inversion models for the three crops. **(A)** Represents maize, **(B)** represents millet, and **(C)** represents sorghum.

**Figure 5:**
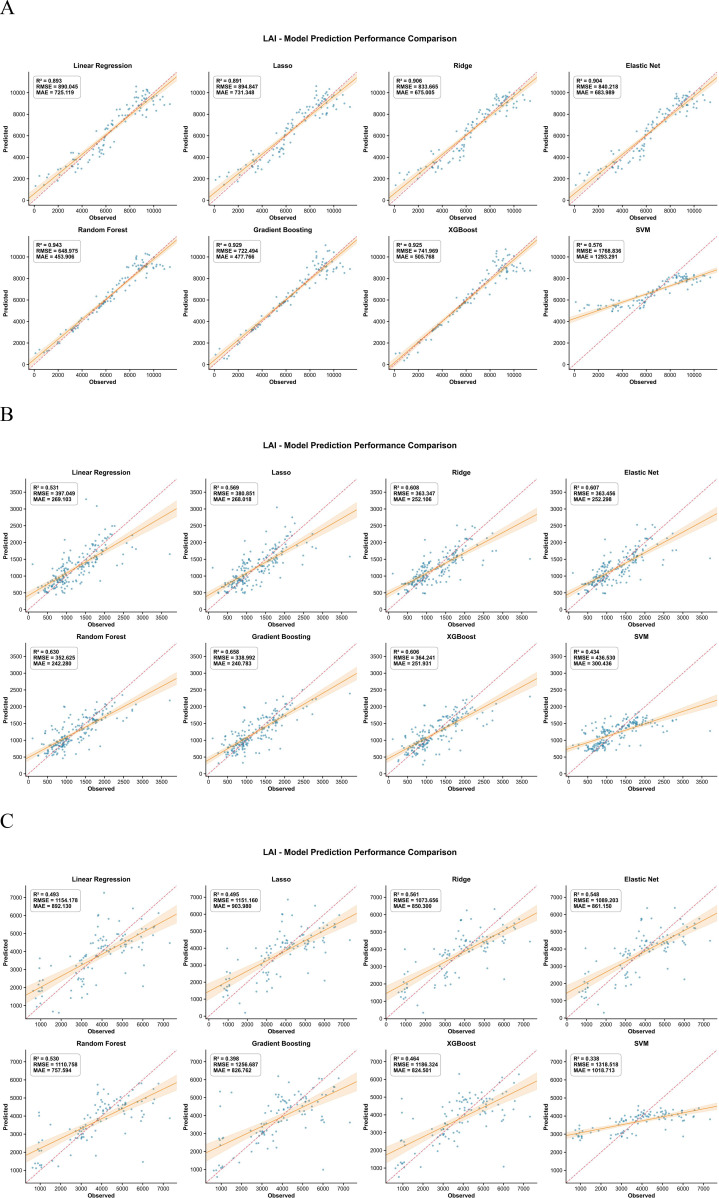
Comparison of predicted versus measured LAI values from the inversion models for the three crops. **(A)** Represents maize, **(B)** represents millet, and **(C)** represents sorghum.

**Figure 6:**
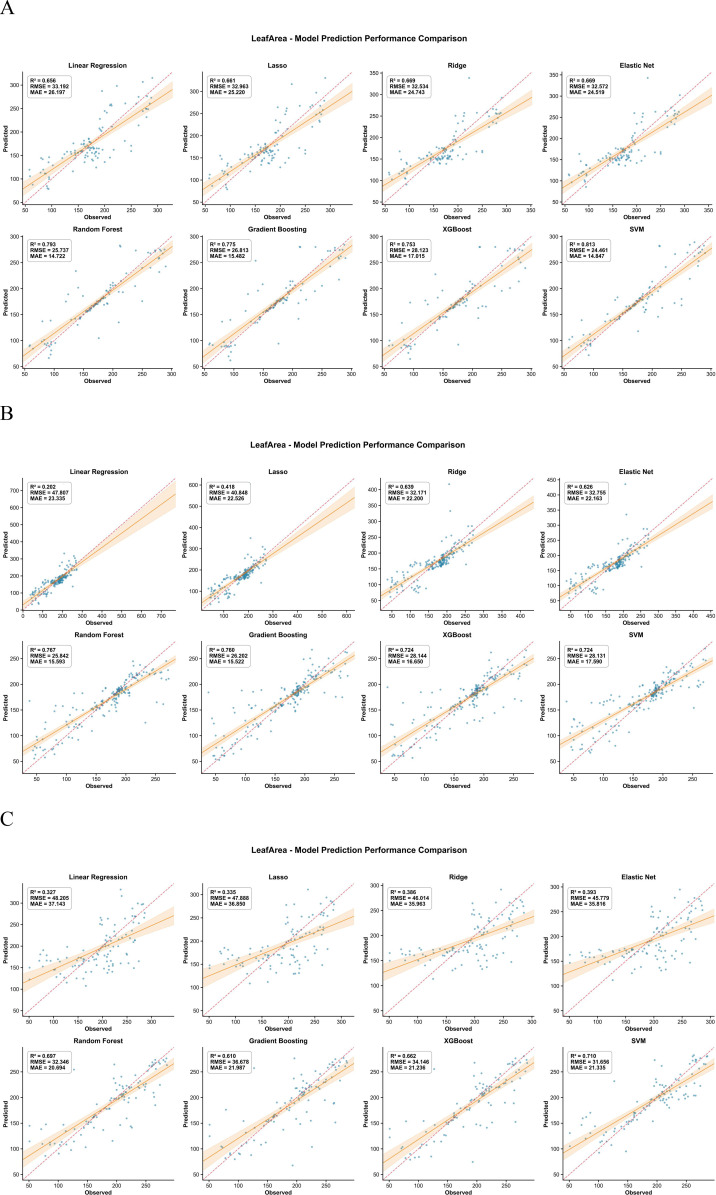
Comparison of predicted versus measured leaf area from the inversion models for the three crops. **(A)** Represents maize, **(B)** represents millet, and **(C)** represents sorghum.

**Figure 7:**
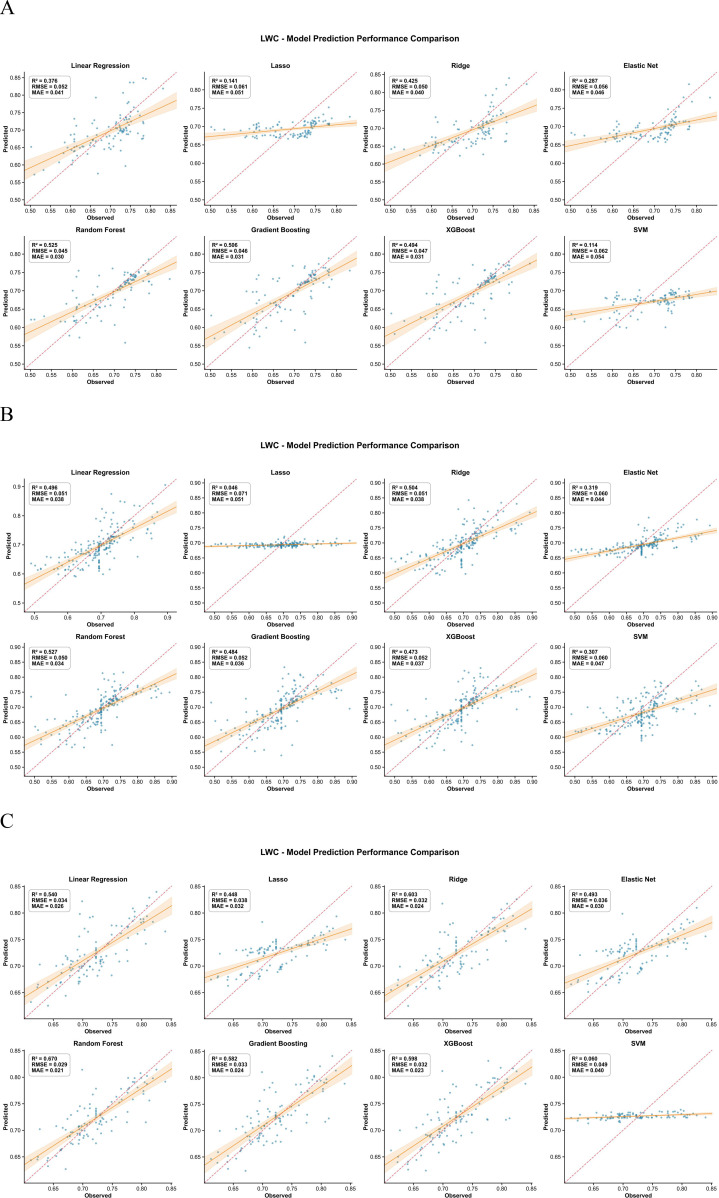
Comparison of predicted versus measured LWC from the inversion models for the three crops. **(A)** Represents maize, **(B)** represents millet, and **(C)** represents sorghum.

For AGB, SPAD, LAI, leaf area and LWC, the inversion models based on UAV multispectral features followed a similar pattern across crops (**Figures 3**–**7**). In maize, most traits reached high R² values (e.g., SPAD: R²=0.944, RMSE = 0.409, MAE = 0.280; LAI: R²=0.943, RMSE = 648.975, MAE = 453.906; AGB: R²=0.723, RMSE = 143.277, MAE = 106.798), and the 1:1 plots showed a narrow scatter around the 1:1 line. In millet, the models achieved intermediate performance (e.g., AGB: R²=0.655, RMSE = 11.914, MAE = 9.297; SPAD: R²=0.726, RMSE = 0.727, MAE = 0.540), whereas sorghum generally showed the lowest agreement between predicted and observed values (e.g., AGB: R²=0.468, RMSE = 112.802, MAE = 79.666; LAI: R²=0.561, RMSE = 1073.656, MAE = 880.300). This ranking is consistent with the field observations: maize stands were more uniform, while sorghum canopies were more heterogeneous, which may increase spectral mixing.

Across traits, AGB and SPAD were easier to predict than LWC, especially in maize. For example, in maize, AGB prediction gave R²=0.723, RMSE = 143.277, MAE = 106.798, while LWC gave R²=0.525, RMSE = 0.045, MAE = 0.030. In millet, AGB (RMSE = 11.914) also outperformed LWC (RMSE = 0.050). This suggests that biomass and leaf greenness have a stronger and more direct expression in the multispectral signal than small changes in water content. The better performance of ensemble algorithms compared with linear regression for most traits (e.g., Random Forest achieved the lowest RMSE and MAE for AGB and LWC across all three crops) implies that the spectral–trait relationships contain interaction effects between bands and vegetation indices that are captured more effectively by tree-based models.

### 3.3 Root biomass inversion models for the three crops

**Figure 8:**
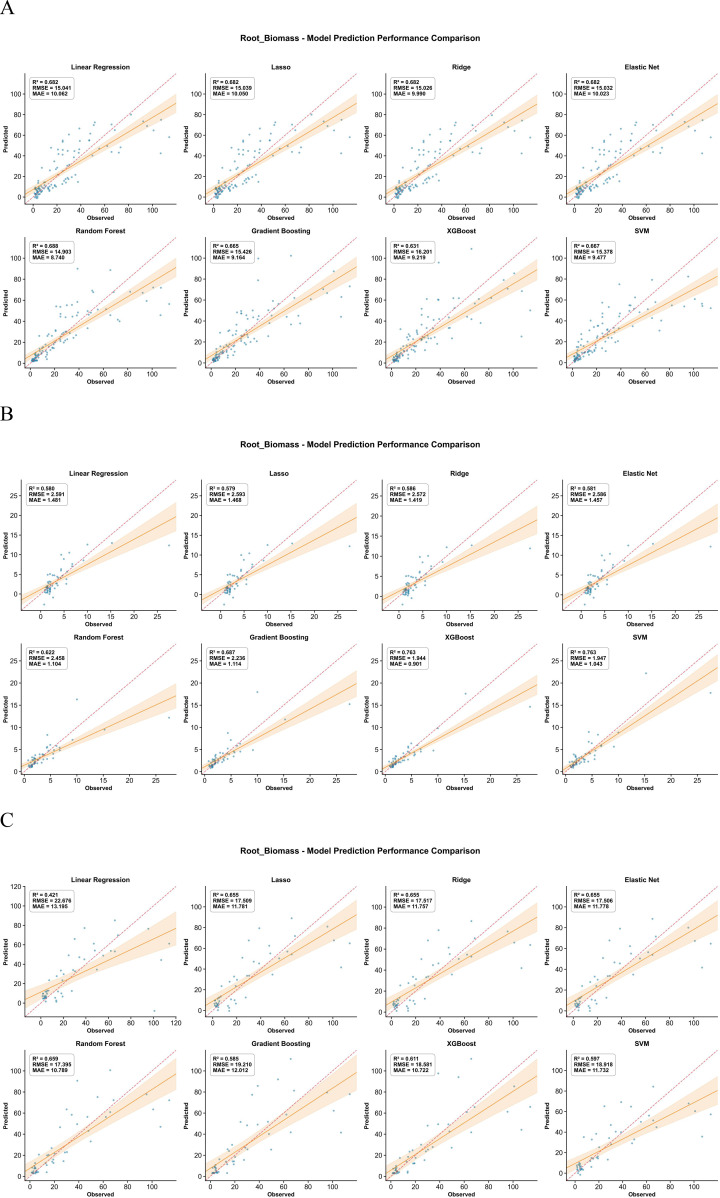
Comparison of predicted versus measured root biomass from the inversion models for the three crops. **(A)** Represents maize, **(B)** represents millet, and **(C)** represents sorghum.

Root biomass inversion based on above-ground phenotypic traits showed clear differences among crops (**Figure 8**). Millet obtained the highest prediction accuracy, with XGBoost providing the best model. Maize achieved moderate accuracy, with Random Forest performing best. Sorghum again showed the lowest accuracy, and all algorithms produced similar but weaker fits than for the other two crops. These differences indicate that the strength of the link between above-ground traits and root biomass is species-specific. In millet, root growth responded more coherently to variations in canopy traits, so the models could capture a larger fraction of the variance. In sorghum, the weaker relationships and greater field heterogeneity reduced model performance, even when using flexible algorithms such as Random Forest and XGBoost.

### 3.4 SHAP analysis of phenotypic features

**Figure 9:**
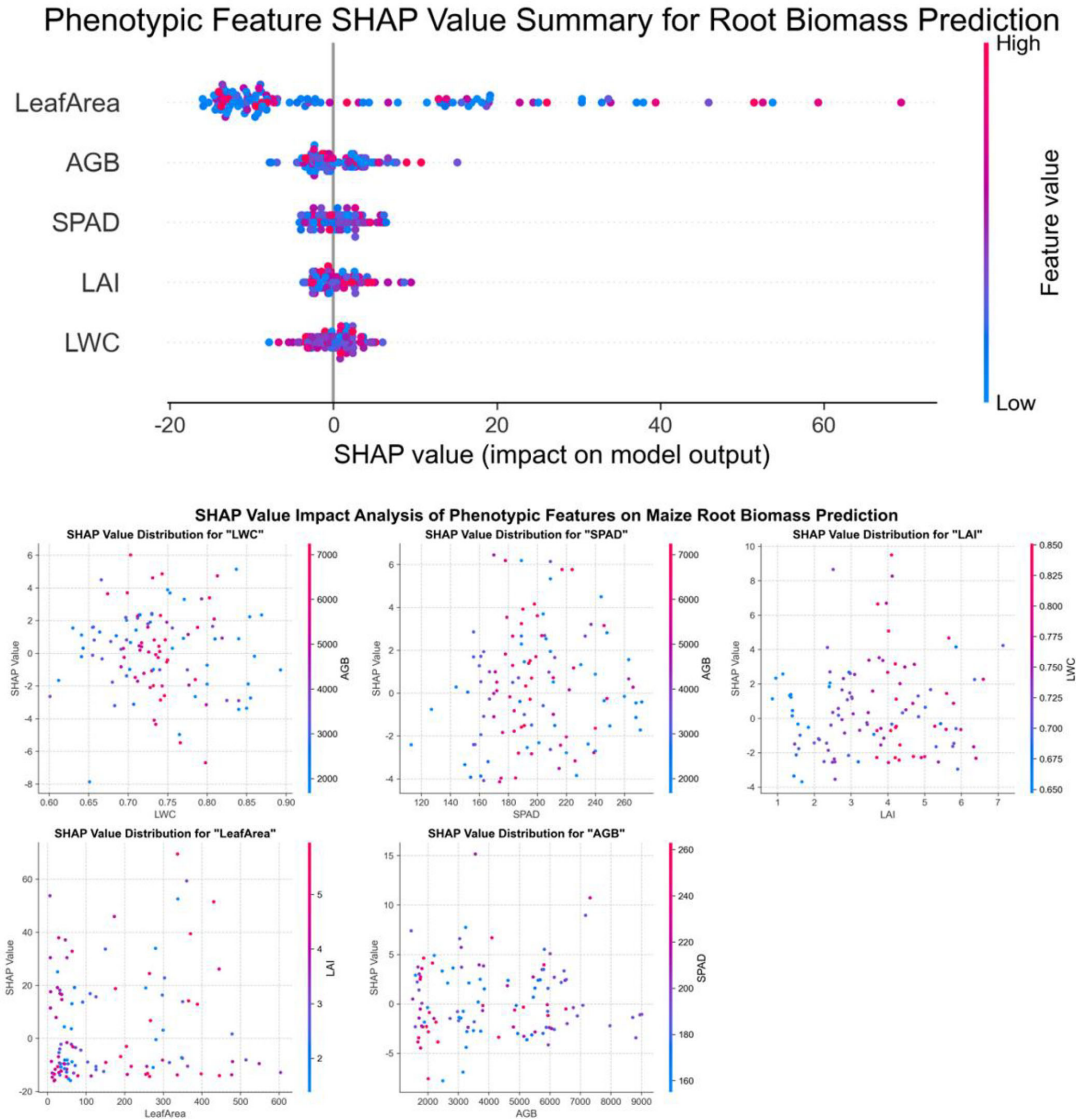
SHAP value analysis of phenotypic features on maize root biomass prediction.

**Figure 10:**
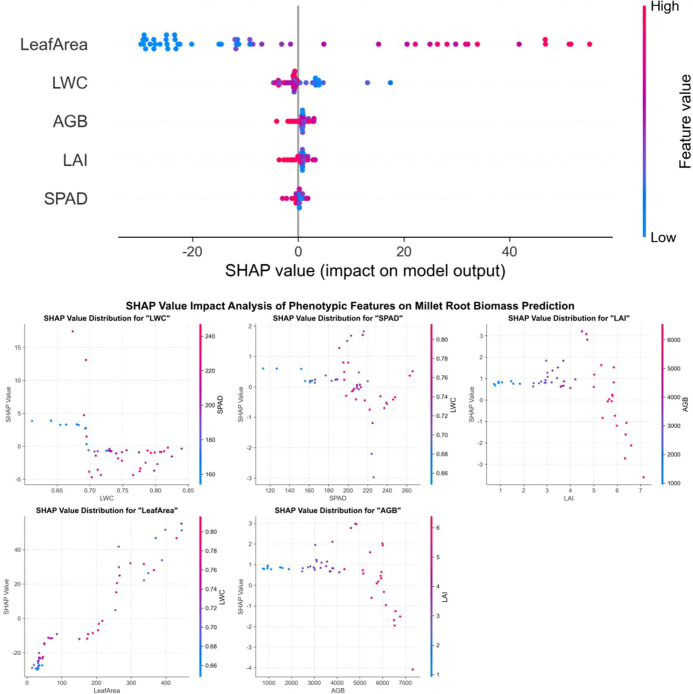
SHAP value analysis of phenotypic features on millet root biomass prediction.

**Figure 11:**
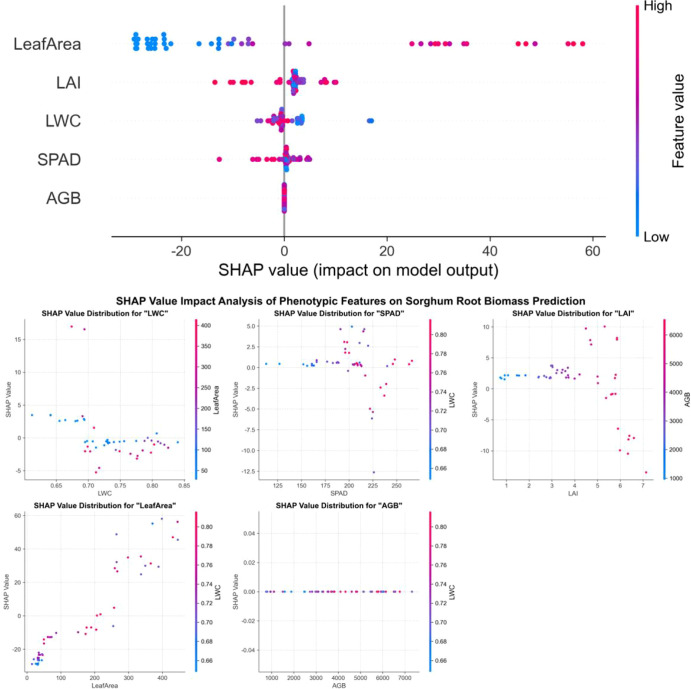
SHAP value analysis of phenotypic features on sorghum root biomass prediction.

SHAP analysis was carried out on the best root biomass model for each crop: Random Forest for maize and sorghum, and XGBoost for millet (**Figures 9**–**11**). Across crops, leaf area was consistently the most influential predictor, with the widest SHAP value range. This suggests that canopy size is an important predictor of below−ground biomass in the model.

The other traits showed distinct roles in different crops. In maize, AGB was the second most influential feature, followed by LAI, while SPAD and LWC had smaller but still positive contributions. In millet, LWC and SPAD were more important than AGB, indicating that water status and leaf greenness convey additional information on root development under dryland conditions. In sorghum, the SHAP values of all traits were more similar to each other, which is consistent with the lower overall model accuracy.

Overall, the SHAP results explain why ensemble models provided better predictions: they can use the combined effect of several traits, with leaf area as the main driver and the other traits adding crop-specific adjustments.

### 3.5 Partial dependence of root biomass on phenotypic traits

**Figure 12:**
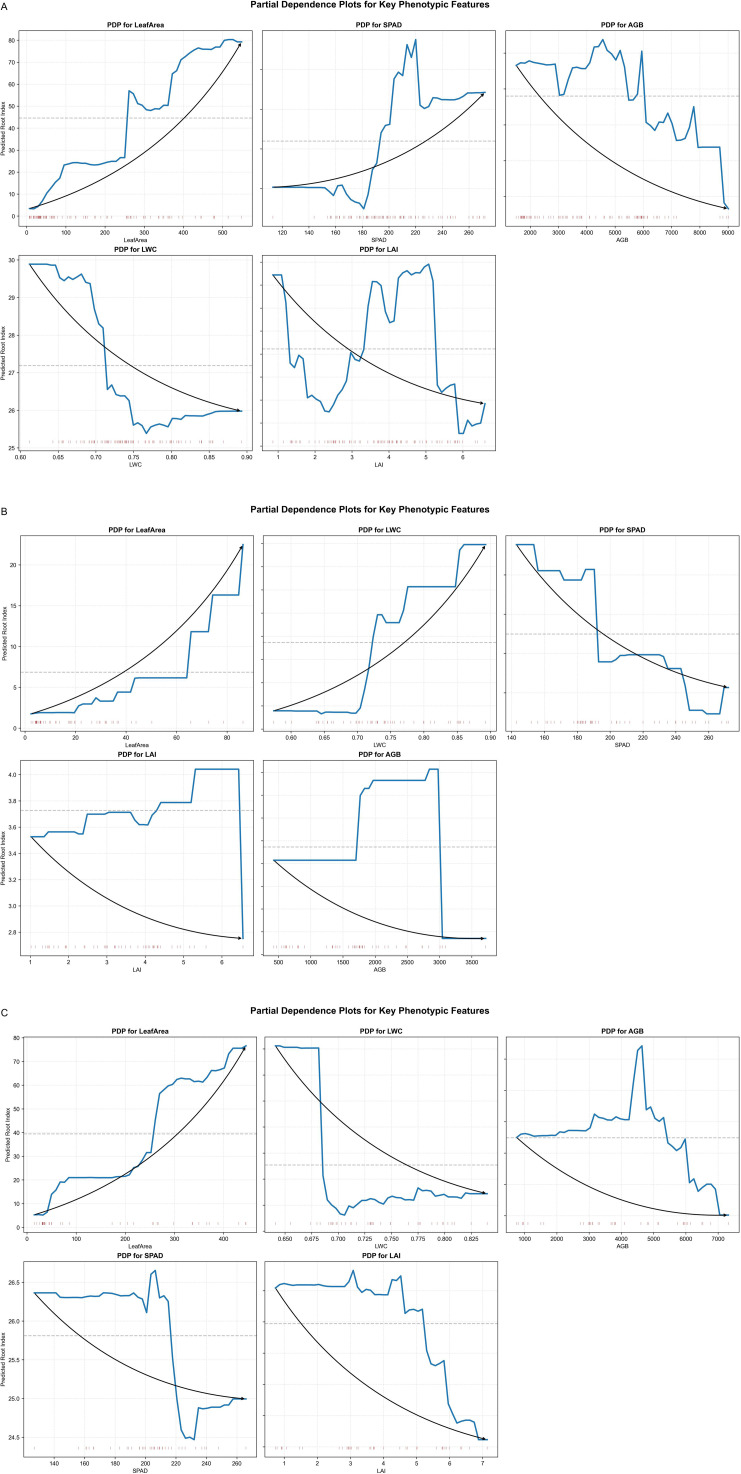
Partial dependence plots for the three crops. **(A)** Represents maize, **(B)** represents millet, and **(C)** represents sorghum.

The Partial dependence plots (**Figure 12**) describe how changes in each phenotypic trait affect the predicted root biomass when other traits are kept fixed. For all three crops, the partial dependence of root biomass on leaf area was almost monotonic, with higher leaf area leading to higher predicted root biomass. This supports the idea that larger canopies tend to be associated with stronger root systems.

For maize, SPAD showed a positive effect within an intermediate range, indicating that moderate to high chlorophyll content is favorable for root growth, while very low values are associated with reduced root biomass. In millet, LWC displayed a clear threshold: below a certain value, increases in LWC had little effect on predicted root biomass, whereas above this threshold the response became strongly positive. In sorghum, several traits exhibited multi-stage responses, with ranges where the marginal effect became weaker or even plateaued, which agrees with the more complex growth pattern of this crop in the field.

Taken together, the SHAP and partial dependence results link the quantitative model outputs to physiological interpretation. Leaf area acts as a general structural indicator of root biomass, whereas LWC, SPAD, LAI and AGB modify this relationship in a crop-specific way, reflecting different strategies of resource allocation under semi-arid conditions.

## 4 Discussion

### 4.1 Model performance and comparison with literature

This study developed root biomass prediction models for three crops using UAV-based multispectral data and machine learning algorithms. Ensemble learning algorithms, particularly XGBoost and RF, achieved the best performance, with R² values of 0.763, 0.688, and 0.659 for millet, maize, and sorghum, respectively. These results are consistent with Li et al (Li et al., 2020), who reported R² = 0.72 using XGBoost for forest AGB prediction. However, direct comparison is complicated by fundamental differences in prediction targets and data structures: forest AGB can be estimated from canopy structural parameters measured by LiDAR, whereas root biomass must be inferred from indirect physiological relationships with above-ground traits. This difference is critical—LiDAR directly measures canopy height and volume, which correlate strongly with woody biomass, while our approach relies on the assumption that above-ground phenotypic traits reflect below-ground development through functional equilibrium. Wang et al (Wang et al., 2016). found RF more stable than SVR and Neural Networks in wheat biomass estimation, supporting our algorithm selection, though their focus on above-ground biomass avoids the inferential challenges inherent in root prediction.

Compared to studies predicting above-ground parameters—such as Zhang et al (Zhang et al., 2022). achieving R² = 0.84 for maize LAI and Wei et al (Wei et al., 2023). achieving R² = 0.87 for wheat AGB—our root prediction accuracy was 15-25% lower. This disparity is not surprising and actually reflects three fundamental challenges in root prediction: (1) root biomass must be inferred indirectly from above-ground traits, whereas LAI and AGB can be directly sensed through spectral reflectance; (2) root systems exhibit greater spatial heterogeneity than above-ground components, introducing additional uncertainty; and (3) these previous studies employed multi-site designs with larger sample sizes, potentially improving model generalization.

This 15-25% reduction in R² quantifies the ‘cost of indirect inference’—the inherent loss of predictive power when estimating an unseen target through its correlates rather than direct observation. This ‘cost of indirect inference’ represents a fundamental trade−off in UAV−based root phenotyping: remote sensing can non−destructively estimate root biomass, but with an inherent loss of predictive accuracy compared to direct measurements. While R² values of 0.66-0.76 explain more than 65% of root biomass variation, which may be considered promising for some applications, the practical utility of this predictive accuracy for tasks such as identifying high− versus low−performing genotypes in breeding programs or guiding variable−rate management in precision agriculture is not yet established. Such applications would require rigorous validation under realistic operational conditions, including assessment of ranking errors and prediction uncertainty. Without this validation, claims of decision−support readiness are premature. Future work should therefore evaluate model performance in targeted use cases, quantify the impact of prediction uncertainty on decision outcomes, and assess whether the observed R² range is sufficient for specific practical tasks. However, these comparisons reveal a fundamental methodological limitation: our models rely on correlative relationships between above−ground and below−ground traits, which may not hold consistently across different environmental conditions or growth stages. Future work should validate these relationships under varying water availability and nutrient regimes to assess model transferability, and should also test the operational suitability of the current prediction accuracy in real−world breeding or management settings.

### 4.2 Crop-specific performance patterns and physiological mechanisms

The Millet’s superior predictability is broadly consistent with the functional equilibrium hypothesis (Brouwer, 1962), which suggests that drought-adapted species may exhibit tighter coordination between above−ground and below−ground resource acquisition. However, the evidence from this study is not sufficient to confirm this mechanism, and the interpretation remains hypothesis−generating rather than conclusive. Zarco-Tejada et al (Zarco-Tejada et al., 2012). documented significant interspecific variation in shoot−root ratio sensitivity to environmental changes across 27 herbaceous species, correlating with ecological strategies. Nevertheless, their controlled environment experiments may not fully capture the complexity of field conditions, where factors such as soil heterogeneity and microclimate variation introduce additional noise. Further studies are needed to test whether the observed predictability differences are indeed driven by the proposed physiological mechanisms.

The intermediate performance of maize can be attributed to its combination of physiological and structural characteristics found in both millet and sorghum. It has moderately coordinated shoot-root relationships (better than sorghum but less tight than millet based on our R² values) and intermediate canopy complexity (taller than millet but more uniform than sorghum’s highly variable canopy structure). This intermediate position in prediction accuracy (R²=0.688) suggests that both physiological coupling and canopy structure contribute roughly equally to model performance—a hypothesis that could be tested by comparing prediction accuracy across cultivars with contrasting plant architecture.

Sorghum’s lower prediction accuracy likely stems from its tall stature and large canopy size, which increase spectral mixing effects and complicate feature extraction from UAV imagery. This interpretation is supported by previous studies showing that canopy structural complexity reduces the accuracy of spectral-based biomass estimates. However, an alternative explanation is that sorghum’s indeterminate growth habit and prolonged vegetative phase create greater temporal variability in shoot-root relationships compared to the more determinate growth patterns of maize and millet. Cairns et al (Cairns et al., 2013). focused on drought tolerance mechanisms in sorghum but did not address remote sensing prediction challenges. Their controlled experiments revealed strong genetic variation in root traits, suggesting that cultivar-specific differences may contribute to prediction uncertainty in field studies like ours. This limitation was not explicitly addressed in our study design, which pooled data across cultivars within each crop species.

The differential algorithm performance across crops also merits discussion. XGBoost’s superior performance in millet may relate to its gradient boosting mechanism being well-suited to subtle feature variations in millet data, while Random Forest’s advantage in maize and sorghum reflects its robustness to noise and outliers. Chen et al (Chen and Guestrin, 2016). emphasized XGBoost’s strength in capturing complex feature interactions, though they noted that performance depends critically on hyperparameter tuning and data structure. Freeman et al (Freeman et al., 2016). confirmed RF’s stability in ecological data processing but also highlighted that its ensemble averaging can obscure interpretable relationships. This trade-off between predictive accuracy and interpretability remains a key challenge in applying machine learning to agricultural systems.

### 4.3 Feature importance and physiological interpretation

SHAP analysis revealed that leaf area was the most important predictor when evaluated within each crop’s model separately, with influence ranges of -25 to 60 for millet, -20 to 60 for maize, and -25 to 60 for sorghum. This finding is consistent with Potgieter et al (Potgieter et al., 2017), who identified leaf area as a key parameter linking photosynthesis and root carbon allocation. Within each crop model, leaf area SHAP values were approximately 2−3 times higher than the second most important features (LWC or AGB), suggesting its dominant role in the model. However, it should be noted that the absolute magnitude of SHAP values is not directly comparable between different crop models, as model structures differ even though the target variable (root biomass) and feature preprocessing were consistent. The observed dominance may be related to the fact that leaf area is a major determinant of photosynthetic capacity, which can influence carbohydrate allocation to roots; however, this interpretation remains correlational and does not imply causation without further experimental validation. This relationship assumes that photosynthate availability, rather than sink strength, is the primary limiting factor for root growth—an assumption that may not hold under all environmental conditions.

LWC in millet showed some variation across its range (e.g., lower contributions between 0.65 and 0.72, and higher contributions above 0.75). However, given the moderate prediction accuracy of the LWC model (R² ~0.52, RMSE ~0.05), these patterns should be interpreted with caution, as they may partly reflect statistical noise rather than a true physiological threshold. The observed trend is qualitatively consistent with Passioura’s (Passioura, 2006) theory, but the evidence from this study is not sufficient to confirm a distinct threshold effect.

Interestingly, maize and sorghum exhibited more linear LWC-root relationships, contrasting with the threshold effect observed in millet. This discrepancy in response patterns may be explained by two factors. First, millet’s superior drought adaptation may make it more sensitive to water status transitions, resulting in sharper threshold responses. Maize and sorghum, being less drought-adapted, may respond more gradually across the water stress gradient. Second, our sampling may not have captured the critical water stress range for maize and sorghum—their thresholds might exist outside the 0.65-0.80 LWC range we observed. This species-specific difference in response patterns may have agronomic relevance, but direct management recommendations are not justified by the current observational/modeling data. The observed patterns suggest that millet root biomass was less associated with higher LWC values in the 0.65−0.72 range, whereas maize and sorghum did not show a clear threshold. Controlled irrigation trials are required to validate these findings before any practical recommendations can be made.

AGB showed stable positive influence in maize and millet but relatively minor contribution in sorghum, reflecting differences in carbon allocation strategies. Reynolds et al (Reynolds and D’Antonio, 1996). observed similar interspecific variation in resource allocation, though their focus on above-ground traits limits direct comparison with root prediction. The weak AGB effect in sorghum aligns with its lower overall prediction accuracy, suggesting that sorghum’s root development may be decoupled from above-ground biomass accumulation—possibly due to its prolonged vegetative growth phase where biomass accumulates in stems rather than being tightly linked to root expansion.

Partial Dependence Analysis suggested crop-specific patterns: SPAD values around 200 were associated with higher contributions in maize, and LAI showed relatively high influence within the range of 2–5. These observed patterns may provide preliminary indications for precision management, but their generalizability across environments and cultivars requires further validation. It should be noted that PDP results can be affected by correlations among input features, especially given our moderate sample size. When variables are correlated, the marginal effects displayed in PDP may not fully reflect true independent contributions. Therefore, these interpretations should be treated as exploratory rather than definitive. To address this limitation, SHAP dependence plots were used to complement PDP analysis, as SHAP values account for feature interactions and provide more robust interpretations.

### 4.4 Spectral technology and vegetation indices

Clarification on the role of VIs and SHAP analysis: In this study, VIs were used as input features to estimate above−ground phenotypic traits (AGB, SPAD, LAI, leaf area, LWC) – referred to as the trait inversion models. The root biomass prediction models, however, used these five estimated (or manually measured) traits as predictors. The SHAP analysis reported in the Results section was performed on the root biomass models using the five traits, not directly on the VIs. Therefore, a full importance ranking of individual VIs is not provided in the Results, as this was not the primary focus of this study. The discussion below on VI performance is based on the trait inversion models, where VIs were used as inputs to estimate above−ground traits. For readers interested in direct VI−to−root biomass prediction or a full VI importance ranking, these represent important directions for future research.

The differential performance of VIs highlights the technical advantages of multispectral technology. The comprehensive application of 13 VIs effectively avoided limitations of single indices, aligning with the multi-index fusion strategy emphasized by Haboudane (Haboudane, 2004). The incorporation of the red-edge band significantly enhanced prediction, particularly for SPAD and LAI, consistent with Gitelson et al (Gitelson et al., 2005). regarding red-edge sensitivity to chlorophyll variation. However, it is important to note that our UAV sensor’s four-band configuration represents a trade-off between spectral detail and operational simplicity. Hyperspectral sensors with hundreds of bands might capture subtler physiological signals but would increase data processing complexity and cost.

The saturation issue of traditional NDVI under high biomass conditions was effectively mitigated by red-edge VIs, validating Zarco-Tejada (Zarco-Tejada, 2000) regarding the advantage of red-edge information. However, their study focused on vineyards with distinct canopy structures, and the transferability of these findings to annual cereal crops warrants careful consideration. The sensitivity differences of various VIs across crops—GNDVI contributing more in millet versus RDVI being more important in sorghum—indicate directions for sensor optimization but also highlight the challenge of developing universal prediction models across species.

### 4.5 Interpretability of machine learning models

Machine learning models employed in this study are fundamentally data-driven “black-box” approaches, differing from mechanistic crop growth models in their underlying philosophy. Process-based models such as APSIM explicitly simulate physiological processes governing biomass partitioning (Kamilaris and Prenafeta-Boldú, 2018), offering transparent cause-effect relationships and theoretical extrapolation capability. However, they require extensive parameterization and may not fully capture field heterogeneity.

In contrast, ML approaches capture empirical relationships directly from observational data without prior mechanistic assumptions. While this limits extrapolation capability, ML excels at handling complex nonlinear interactions in high-dimensional spaces. Recent literature advocates hybrid approaches integrating both paradigms (Zhang et al., 2023). In this study, we addressed interpretability limitations through SHAP and PDP analyses, revealing patterns consistent with functional equilibrium theory. The agreement between data-driven findings and mechanistic understanding suggests that ML models captured biologically meaningful relationships, partially bridging the gap between prediction and interpretation.

### 4.6 Methodological limitations

Several methodological limitations warrant consideration. First, the single-site experimental design limits spatial generalizability. Our models were developed using data from one location with specific soil type, climate, and management practices. Lobell (Lobell, 2013) emphasized that regional variation strongly affects model transferability, and our models require validation across diverse agroecological zones before operational deployment. The limited plot size (3 plots per crop) and spatial extent may not adequately capture landscape-level heterogeneity in soil properties, topography, and microclimate that influence root-shoot relationships. Consequently, the model’s transferability to other soil types (e.g., clay vs. sandy loam) or climatic conditions (e.g., humid vs. semi−arid) cannot be assumed without dedicated validation. Future work should explicitly test the model across edaphic and climatic gradients to assess its robustness and define its operational domain.

Second, the constrained spectral range of our four-band multispectral sensor may have limited information capture. While cost-effective and operationally practical, this configuration lacks the spectral resolution to detect subtle physiological signals potentially related to root function, such as xanthophyll cycle pigments or water absorption features. Integration of hyperspectral imaging or complementary technologies like LiDAR for canopy structural characterization could improve prediction accuracy.

Third, feature collinearity among vegetation indices presents interpretability challenges. Many VIs are mathematically related and derived from the same spectral bands, potentially inflating their apparent importance in SHAP analysis. Moreover, SHAP explanations can become unstable when features are highly correlated, as the contribution of a given predictor may be redistributed among correlated variables. While tree-based algorithms like RF and XGBoost are relatively robust to collinearity, the biological interpretation of feature importance should be approached cautiously. Variance Inflation Factor analysis could quantify collinearity severity and guide feature selection in future iterations.

Fourth, temporal generalizability remains uncertain. Our models were trained on data from specific growth stages within a single growing season. Yang et al (Yang et al., 2017). demonstrated that shoot-root relationships change dynamically throughout the growth cycle, and our static models may not capture these temporal dynamics. Development of dynamic prediction models incorporating phenological stage as an explicit input variable represents an important research direction. This temporal limitation is particularly relevant given the spatial limitation discussed earlier. Together, they suggest that our models currently represent “snapshot” predictions—accurate for specific times and places but requiring recalibration for broader application. The challenge ahead is developing models that maintain accuracy across both space (different sites, soils, climates) and time (different growth stages, years, seasons). Hybrid approaches combining mechanistic understanding of shoot-root dynamics with machine learning flexibility may offer a path forward. In particular, it should be emphasized that the allometric relationships between above−ground and root biomass are highly dependent on soil fertility and water availability. Since our study was conducted under rainfed conditions with specific soil properties, the current model is unlikely to be directly transferable to irrigated fields or those with high levels of fertilization without additional calibration and validation.

Fifth, formal statistical tests comparing algorithm performance were not conducted. While consistent ranking of algorithms across cross-validation folds suggest reliable differences, statistical tests such as Friedman test or paired t-tests would provide formal significance assessments. Additionally, prediction accuracy for sorghum (R² = 0.659) remains sub-optimal and might be improved through additional spectral bands or integration of other remote sensing modalities.

Sixth, no systematic hyperparameter tuning was performed. The parameters were fixed based on literature and preliminary tests. While this approach allowed a fair comparison of algorithms under consistent configurations, it may not represent the optimal performance each algorithm can achieve. Future studies could employ rigorous tuning strategies (e.g., grid search with cross−validation) to further improve prediction accuracy.

Seventh, standard 5−fold cross−validation was used without accounting for potential spatial and temporal dependencies in the dataset. Because repeated measurements were taken from the same experimental plots across multiple growth stages, samples from the same plot or adjacent dates could appear in both training and validation folds, potentially leading to optimistic performance estimates due to information leakage. A more rigorous approach would be leave−one−plot−out or leave−one−date−out cross−validation, which ensures that all samples from a given plot or date are held out together. We acknowledge this limitation and recommend that future studies adopt spatially or temporally grouped cross−validation strategies to obtain less biased estimates of model generalizability. In our current implementation, we used random fold creation without grouping, which may have contributed to optimistic performance estimates, particularly for LAI and SPAD predictions in maize. Readers are advised to interpret the high R² values with caution.

Eighth, sampling intensity (3 plants per plot per date) may be insufficient to capture within-plot spatial variability in root biomass. Root systems exhibit substantial small-scale heterogeneity driven by soil structure, nutrient patches, and inter-plant competition. More intensive sampling or integration with proximal sensing technologies for continuous monitoring would strengthen future model development. The combination of high spatial heterogeneity of root systems with this limited sample size introduces additional uncertainty into our estimates, which may affect both the precision of measured root biomass and the generalizability of the models. Future studies should consider geostatistical approaches or increased sampling densities to better quantify and account for this uncertainty.

Ninth, the two−stage modeling approach may propagate prediction errors from the first stage (trait inversion) to the second stage (root biomass prediction). The validation statistics reported in **Figures 3**–**8** reflect per−stage uncertainties but not the combined uncertainty of the entire workflow. We did not formally propagate the prediction errors from the trait inversion models to the root biomass models. Consequently, the reported performance metrics may underestimate the total uncertainty associated with multi−stage modeling. Future work should employ error propagation techniques (e.g., Monte Carlo simulation or bootstrap) to quantify the combined uncertainty more rigorously.

Tenth, we did not perform a systematic analysis of direct radiometric (spectral−root) correlations. While our two−stage approach focuses on physiologically interpretable traits, a direct spectral−root analysis could identify simpler proxy models for root growth. Future studies should systematically evaluate the correlations between spectral bands/vegetation indices and root biomass to explore such simplified approaches.

Eleventh, the test set size was relatively small (~27 samples per crop) due to the 80/20 train−test split. While cross−validation stability suggests reliable performance, a small test set may lead to higher variability in performance estimates. Future studies should employ nested cross−validation or repeated random splits to provide more robust stability assessments.

Finally, feature scaling was not applied before model training. While tree−based models are scale−invariant, SVR and regularized linear models (Lasso, Ridge, Elastic Net) are sensitive to feature magnitudes. The lack of scaling may have affected their performance. Future studies should apply standardization (e.g., z−score) for such algorithms.

### 4.7 Practical implications and future directions

Despite these limitations, this study provides a novel approach for non-destructive, high-efficiency root monitoring across large areas, addressing a critical gap in precision agriculture (Mulla, 2013; Huang et al., 2018). The root monitoring capability developed here can inform irrigation and fertilization decisions, though operational implementation requires further validation of model robustness across environments and cultivars.

Revisiting the comparisons detailed in Section 4.1, while our root prediction accuracy (R²=0.66-0.76) is 15-25% lower than above-ground trait predictions, this study demonstrates that remote prediction of root biomass is feasible across multiple C4 crop species. The crop-specific patterns we observed—millet’s superior predictability, sorghum’s challenges with canopy complexity—provide a roadmap for where this technology can be deployed immediately (drought-adapted crops with simple canopies) versus where additional development is needed (tall crops with complex architecture).

Given that leaf area was the dominant predictor in our SHAP analysis (2−3 times higher importance than other traits), a simpler univariate model using only leaf area may achieve comparable predictive performance for root biomass. While we did not test such a model in this study, future work should systematically compare the full multi−trait model with a leaf−area−only model to assess whether the additional traits (LWC, SPAD, AGB, LAI) provide meaningful improvement in predictive accuracy, uncertainty, and generalizability across different crops and environments.

Future research should focus on multi-site validation, integration of additional remote sensing technologies, development of dynamic models across growth stages, and explicit quantification of prediction uncertainty for decision support applications. In addition, a systematic comparison between the full multi−trait model and a leaf−area−only model would help determine whether the additional traits (AGB, SPAD, LAI, LWC) provide meaningful improvement in predictive accuracy. Furthermore, while we used Leaf Water Content (LWC) as a measure of plant water status in this study, Equivalent Water Thickness (EWT) — which normalizes water content by leaf area — is known to have a more direct relationship with leaf spectral reflectance. Future studies should compare LWC and EWT for UAV−based water status estimation to determine which metric offers better predictive performance for root−related traits. In addition, direct spectral−root models (using spectral bands or vegetation indices as direct predictors of root biomass) should be compared with the two−stage approach proposed here to determine whether the additional physiological interpretability justifies the increased complexity.

## 5 Conclusion

This study aimed to evaluate whether UAV-based multispectral phenotyping can serve as a non-destructive approach for estimating root biomass in dryland cereal crops. The results demonstrate that vegetation indices (VIs) derived from UAV imagery contain sufficient information to support root biomass prediction within a two-stage modeling framework.

Across the three species examined, leaf area consistently emerged as the most informative predictor of below-ground biomass, indicating a stable association between canopy structural development and root system growth. Other phenotypic variables, including LWC, SPAD, AGB, and LAI, contributed to prediction with crop-specific patterns, reflecting differences in physiological strategies under semi-arid conditions.

Millet’s superior predictability (R² up to 0.763) reflects its simple canopy and fibrous root system, whereas sorghum’s lower accuracy (R² as low as 0.468) is likely due to its heterogeneous canopy increasing spectral mixing. The four−band sensor’s limited spectral resolution may have exacerbated this issue. Our results are broadly consistent with Potgieter et al (Potgieter et al., 2017). but extend to a cross−species field comparison.

Compared with direct spectral−root models from the literature (typically achieving R² = 0.50–0.70), our two−stage approach achieves comparable accuracy (R² = 0.66–0.76) while offering physiological interpretability. Direct models cannot identify which above−ground traits drive root variation, whereas our SHAP analysis reveals leaf area as the dominant predictor. This interpretability is a key advantage of the two−stage framework.

Among the modeling methods tested, ensemble algorithms showed numerically improved predictive performance over linear models, indicating a consistent advantage in capturing nonlinear aspects of shoot–root interactions. Statistical significance was not tested. The SHAP and PDP analyses provided interpretable evidence supporting the relevance of the phenotypic variables used, reinforcing the biological plausibility of the model outputs.

Beyond methodological performance, the findings highlight the practical value of integrating UAV phenotyping into root-focused studies. By reducing reliance on labor-intensive destructive sampling, this approach has the potential to support large-scale screening of below-ground traits, improve monitoring efficiency in dryland agriculture, and facilitate the development of management strategies that consider both canopy and root dynamics.

Future research should extend this framework across additional seasons, environments, and sensing modalities to evaluate robustness and generalizability. Doing so will further clarify the potential of UAV-assisted phenotyping as a practical tool for advancing root-centered crop research in water-limited ecosystems.

## Data Availability

The original contributions presented in the study are included in the article/supplementary material. Further inquiries can be directed to the corresponding author.
